# Are the determinants of the progression to type 2 diabetes and regression to normoglycemia in the populations with pre-diabetes the same?

**DOI:** 10.3389/fendo.2022.1041808

**Published:** 2022-10-07

**Authors:** Zeinab Alizadeh, Hamid Reza Baradaran, Karim Kohansal, Farzad Hadaegh, Fereidoun Azizi, Davood Khalili

**Affiliations:** ^1^ Department of Epidemiology, School of Public Health, Iran University of Medical Sciences, Tehran, Iran; ^2^ Ageing Clinical and Experimental Research Team, Institute of Applied Health Sciences, School of Medicine, Medical Sciences and Nutrition University of Aberdeen, Aberdeen, United Kingdom; ^3^ Endocrine Research Center, Institute of Endocrinology and Metabolism, Iran University of Medical Sciences, Tehran, Iran; ^4^ Prevention of Metabolic Disorders Research Center, Research Institute for Endocrine Sciences, Shahid Beheshti University of Medical Sciences, Tehran, Iran; ^5^ Endocrine Research Center, Research Institute for Endocrine Sciences, Shahid Beheshti University of Medical Sciences, Tehran, Iran; ^6^ Department of Biostatistics and Epidemiology, Research Institute for Endocrine Sciences, Shahid Beheshti University of Medical Sciences, Tehran, Iran

**Keywords:** normoglycemia, pre-diabetes, type 2 diabetes, Cardiometabolic disorders, progression, regression

## Abstract

**Background:**

We aimed to determine the predictors of regression to normoglycemia and progression to diabetes among subjects with pre-diabetes in a single model concurrently.

**Methods:**

The present study included 1329 participants aged 20 to 70 years with prediabetes from the population-based cohort of the Tehran Lipid and Glucose Study, with a 10-year follow-up. Glycemic status at follow-up was categorized as regression to normoglycemia: fasting plasma glucose [FPG] of <5.55 and 2h-plasma glucose [PG] of <7.77 mmol/L, and not taking antidiabetic medications. Glycemic status at follow-up was categorized as progression to diabetes: FPG ≥7 or 2h-PG of ≥11.1 mmol/L, or taking antidiabetic medications. Glycemic status determined whether the patients remained in prediabetes category (isolated impaired fasting glycaemia [iIFG] [(5.55≤FPG<7 and 2h-PG<7.77 mmol/L); isolated impared glucose tolarence [iIGT] (7.77 ≤ 2h-PG<11.1 and FGP<5.55 mmol/L)]. With prediabetes as a reference, multinomial logistic regression was utilized to identify the determinants of glycemic changes.

**Results:**

Approximately 40% of participants returned to normoglycemia (n = 578), and similar percentage of participants progressed to diabetes (n = 518). Based on the multivariable multinomial model, regression to normoglycemia was associated with age (relative risk ratio [RRR] = 0.97; 95% CI, 0.95-0.99), female sex (RRR = 1.72; 95% CI, 1.18-2.50), high education level of ≥12 years (RRR = 2.10; 95% CI, 1.19-3.70), and combined IFG/impaired glucose tolerance (IGT) versus IFG (RRR = 0.45; 95% CI, 0.29-0.70). The risk of progression to diabetes increased with body mass index (RRR = 1.10; 95% CI, 1.05-1.15), waist circumference (RRR = 0.97; 95% CI, 0.96-0.99), positive familial history of diabetes (RRR = 1.62; 95% CI, 1.07-2.45), and combined IFG/IGT versus IFG (RRR = 2.54; 95% CI, 1.71-3.77).

**Conclusion:**

A small percentage of patients with prediabetes remain in this condition, but the majority go on to develop diabetes or regress to normoglycemia. Both directions had distinct predictors.

## Introduction

Prediabetes is understood to be a critical metabolic stage in the onset of diabetes and its complications. In prediabetes, glucose levels are higher than normal but not yet at the threshold for diabetes. The number of people with prediabetes is rapidly rising in all countries around the world. In terms of disease burden, high fasting plasma glucose (FPG) ranked fifth in 2017. Globally, 352 million adults (7.3%) had prediabetes, and that number was projected to rise to 587 million (8.3%) by the year (1) 2045. Rates of progression to diabetes and regression to normoglycemia from prediabetes have been reported differently in previous studies. Every year, 5% to 10% of those with prediabetes may develop diabetes, while the same number may develop normoglycemia. According to the American Diabetes Association expert panel, 70% of people with prediabetes will eventually develop diabetes (2).

Regression ranged from 33% to 59% within 1 to 5 years’ follow-up in 47 studies (3). Clinical studies have confirmed that lifestyle modification programs focusing on consuming a healthier diet and engaging in more physical activity can lower the risk of developing diabetes. Reversion from prediabetes to normoglycemia is associated with improving a range of cardiovascular risk factors (1).

In this study, our objective was to determine the predictors of the regression to normoglycemia and progression to diabetes among adults with pre-diabetes in a single model simultaneously using a population-based cohort study with ten years of follow-up.

## Methods

### Study design and population

**Figure 1:**
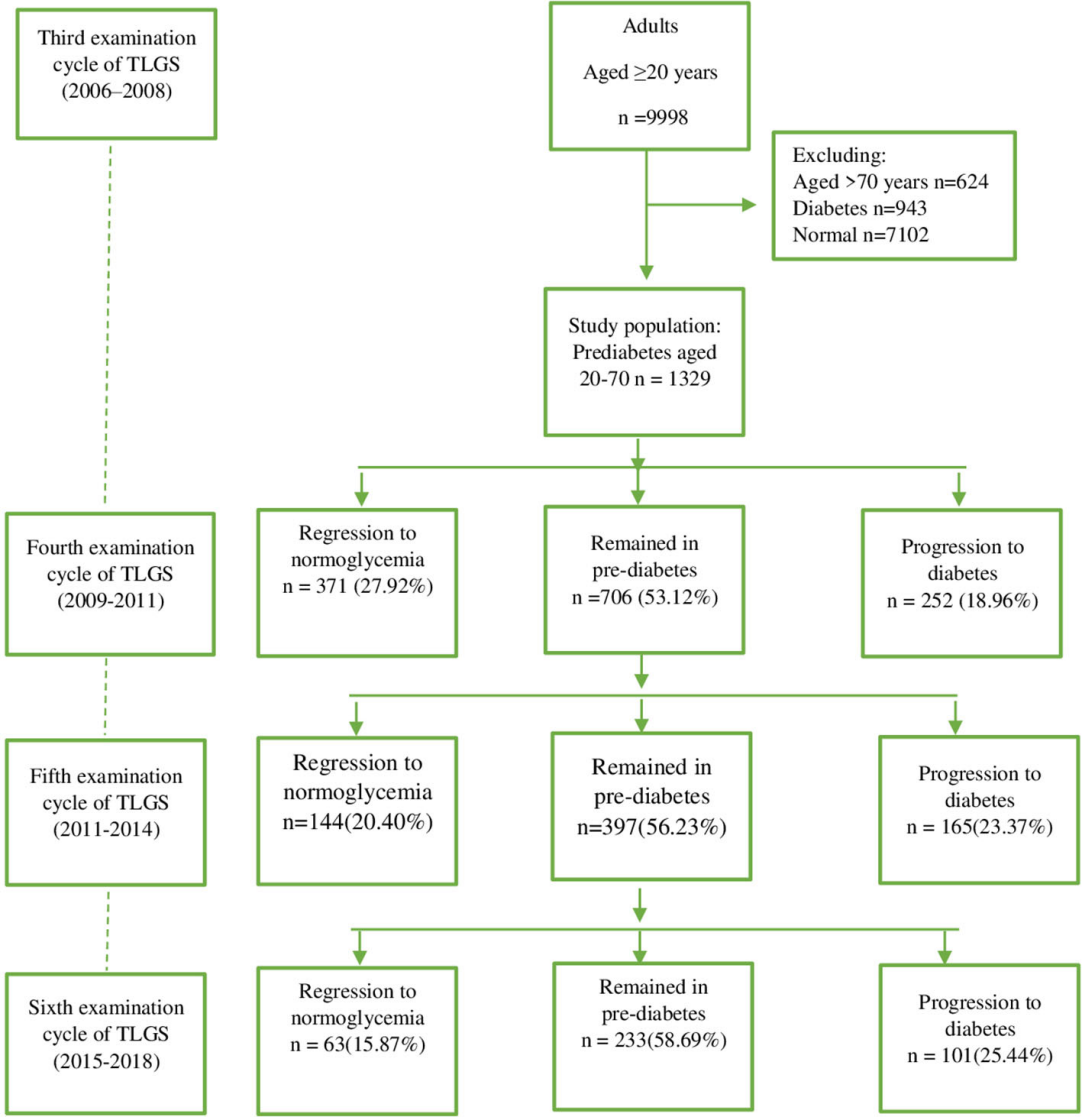
Flow diagram describing the study population.

The Tehran Lipid and Glucose Study (TLGS)—the first community-based large-scale, long-term cohort study in Iran—was designed (4) in 1998. The TLGS was initiated in 1999 to investigate noncommunicable disease (NCD) and its associated risk factors or determinants among a representative population of Tehran. The baseline measurement was conducted between February 1999 and August 2001. In this study, those who were 3 years old or older and residing in the District 13 of Tehran were considered the reference population. Currently, the project is in its seventh phase. A total of 15,005 people aged 3 and older were recruited during the baseline data collection phase of the project (1999-2001), and they were examined for NCD risk factors—a procedure that is repeated every 3 years following the standardized protocol. Data for this study were taken from the third examination cycle (n = 9998). We considered adults aged 20-70 years with the diagnosis of prediabetes as the study population. So, those with the diagnosis of diabetes, defined as FPG ≥7 or 2h-PG ≥11.1 mmol/L or taking anti-diabetic medications (n=943), and those with normoglycemia (FPG<5.55 and 2h-PG<7.77 mmol/L and not taking anti-diabetic medications) were excluded. Because of comorbidities in people over 70 years old, we excluded these participants as well (n=624). The flowchart in **Figure 1** shows the description of the study population. Finally,1329 participants with prediabetes aged 20 to 70 years remained eligible and were observed for 10 years in the current analysis. The study was reviewed and approved by the ethics committee of Shahid Beheshti University of Medical Sciences (Ethics approval reference number: IR.SBMU.ENDOCRINE.REC.1400.113).

### Measurements

Each interview was conducted through a structured questionnaire to collect demographic data, education level, smoking status, medication use, family history of diabetes, history of cardiovascular disease (CVD), or family history of CVD. The mercury column sphygmomanometer was used to measure the systolic and diastolic blood pressure (SBP, DBP), and the mean of 2 consecutive measurements on the same arm after at least 5 minutes of seated rest in a chair was calculated. The standard measurement techniques were used to determine the body weight, waist sizes, and height. A venous sample was taken between 7:00 AM and 9:00 AM after 12 to 14 hours of fasting for laboratory testing, and all samples were analyzed in the TLGS research laboratory on the day of blood sampling. The details and protocols of the TLGS clinical measurements were published elsewhere (5).

### Definition of variables

At the baseline or each examination, participants were classified as (1) having diabetes (fasting plasma glucose [FPG] ≥7 or 2h-PG ≥11.1 mmol/L, or taking antidiabetic medications), (2) normoglycemia (FPG <5.55 and 2h-PG<7.77 mmol/L and not taking antidiabetic medications), and (3) as prediabetes (isolated impared fasting glucose [IFG] [5.55≤FPG<7 and 2h-PG<7.77 mmol/L]; isolated impared glucose tolerance [IGT] [7.77 ≤ 2h-PG<11.1 and FGP<5.55 mmol/L] and combined IFG/IGT [5.55≤FPG<7 and 7.77 ≤ 2h-PG<11.1 mmol/L]). If any participant’s first-degree relatives had type 2 diabetes, it was regarded as having a positive family history of the disease. Body mass index (BMI) was calculated as weight in kilograms divided by squared height in meters. Smoking status was categorized as follows: current, former, and nonsmokers. Furthermore, participants were divided into 3 groups based on their length of education: 0 to 5, 6 to 12, and >12 years. The outcome of our study was evaluated by whether patients developed diabetes or normoglycemia for the first time or maintained prediabetes during our follow-up. The event date was taken into account as the point at which the person first experienced normoglycemia or diabetes and last experienced prediabetes; for those without a normoglycemia or diabetes event, the most recent follow-up time was taken into account.

### Statistical analysis

Baseline characteristics were summarized as mean and standard deviation for continuous variables and frequencies (%) for categorical variables. The predictive mean matching method and a 5-time imputation with 50 iterations were used to perform multiple imputations for missing data at the baseline (up to 12.4% for different variables) and follow-up (up to 29% in separate examinations). One-way analysis of variance and chi-square tests were used to compare continuous and categorical variables between the groups. Multinomial logistic regression was performed to calculate the relative risk ratio (RRR) and 95% CI for the considered risk factors. First, a univariable analysis of potential predictors were performed that included age, sex, BMI, SBP, DBP, use of antihypertensive drugs, use of antihyperlipidemic drugs, positive familial history of type 2 diabetes, waist circumference, glycemic status, total cholesterol (TC), triglycerides (TG), high-density lipoprotein cholesterol (HDL-C), personal history of CVD, familial history of CVD, smoking status, and education level. For the next step, variables presenting *P* <.2 were included in the multivariable model— all variables were significant in one or both outcomes. The interaction of the selected variables with age and sex was assessed using the likelihood ratio test (LR test). Since the LR test was not significant, the interaction terms were not entered into the multivariable model. We also checked the interaction between type of prediabetes (IFG/IGT) and other predictors and did not find a significant interaction. BMI had a strong correlation with waist circumference (*r* = 0.78), thus,a sensitivity analysis was performed. Separate models for weight and waist circumference were developed. (see **Supplementary Tables**). Continuous variables were centralized to ease the interpretation of intercept terms. An RRR> 1 suggests a higher risk for regression in the case of regression to normoglycemia, which is a favorable outcome. In the case of a progression to diabetes, an RRR >1 indicates a higher risk of progression, which is an unfavorable outcome. Statistical significance was set at *P* <.05. All analyses were performed using STATA Version 16 (Stata Corp).

## Results

We assessed 1329 people with prediabetes between 2006 and 2018, and the median follow-up time was 10 years (interquartile range, 0.9 years).

Baseline clinical characteristics and laboratory data according to transition status during follow-up are presented in **Table 1**. The BMI, systolic blood pressure, diastolic blood pressure, waist circumference, FPG, 2h-PG, TC, TG, likelihood of using a drug to treat hyperlipidemia, positive family history of type 2 diabetes, and dyslipidemia were all higher in participants who had progressed to diabetes. However, those who regressed to normoglycemia had more favorable values. No significant differences across categories were observed regarding CVD history, familial history of CVD, and smoking status. Overall, approximately 40% of participants (n = 578) (men, 43.03; women, 43.86) returned to normoglycemia, and 40% of participants (n = 518) (men, 36.56; women, 40.89) progressed to diabetes (see **Figure 1** and **Table 2**).

**Table 1:** Baseline characteristics of participants according to transition status^a^.

Variables	Total Participants (N = 1329)	Remained in Prediabetes (n = 233)	Regression to Normoglycemia (n = 578)	Progression to Diabetes (n = 518)	*P*
**Age, years**	50.01 ± 12.11	52.46 ± 10.69	47.27 ± 13.20	51.97 ± 10.77	<.001
**Sex, female**	741 (55.76)	113 (48.50)	325 (56.23)	303 (58.49)	.037
**BMI, Kg/m** ^2^	29.43 ± 4.88	28.96 ± 4.30	28.64 ± 4.70	30.53 ± 5.12	.003
**SBP, mmHg**	121.29 ± 18.41	122.70 ± 17.30	117.80 ± 17.76	124.56 ± 18.95	<.001
**DBP, mmHg**	77.32 ± 10.30	77.42 ± 10.36	75.69 ± 9.84	79.10 ± 10.49	<.001
**Antihypertensive drugs**	98 (7.37)	19 (8.15)	30 (5.19)	49 (9.46)	.023
**Antihyperlipidemic drugs**	79 (5.94)	19 (8.15)	24 (4.15)	36 (6.95)	.043
**Familial History of T2DM**	282 (21.22)	41 (17.60)	106 (18.34)	135 (26.06)	.003
**Waist circumference, cm**	97.15 ± 11.42	97.52 ± 10.66	94.97 ± 11.64	99.42 ± 11.05	<.001
**FPG, mmol/L**	5.62 ± .52	5.63 ± .47	5.44 ± .50	5.81 ± .50	<.001
**2h-PG, mmol/L**	7.61 ± 1.75	7.46 ± 1.74	7.17 ± 1.66	8.18 ± 1.70	<.001
**TC, mmol/L**	5.26 ± 1.05	5.26 ± .99	5.18 ± 1.01	5.36 ± 1.11	.0171
**TG, mmol/L**	2.13 ± 1.27	2.16 ± 1.08	1.99 ± 1.33	2.27 ± 1.26	.0008
**HDL-C, mmol/L**	1.04 ± .25	1.02 ± .23	1.06 ± .27	1.02 ± .23	.0147
**Personal history of CVD**	59 (4.44)	11 (4.72)	22 (3.81)	26 (5.02)	.606
**Familial history of CVD**	121 (9.10)	20 (8.58)	53 (9.17)	48 (9.27)	.953
**Smoking**					.484
**Former**	134 (10.08)	26 (11.16)	62 (10.73)	46 (8.88)	
**Current**	152 (11.44)	28 (12.02)	72 (12.46)	52 (10.04)	
**Education**					<.001
**<6 years**	496 (37.32)	103 (44.21)	182 (31.49)	211 (50.85)	
**6-11 years**	624 (46.95)	106 (45.49)	280 (48.44)	238 (38.75)	
**≥12 years**	209 (15.73)	24 (10.30)	116 (20.07)	69 (13.32)	

^a^Data are presented as mean ± SD or n (%). One-way analysis of variance and Chi-Square tests were used to compare continuous and categorical variables between the groups. BMI, body mass index; CVD, cardiovascular disease; DBP, diastolic blood pressure; FPG, fasting plasma glucose; HDL-C, high-density lipoprotein cholesterol; SBP, systolic blood pressure TC, total cholesterol; TG, triglyceride; T2DM, type 2 diabetes; 2h-PG, 2-hour plasma glucose.

**Table 2:** Cumulative incidence of regression to normoglycemia and progression to diabetes by sex during a 10-year follow-up among people with prediabetes*^a^*.

	Total	Male	Female
**Regression to normoglycemia**	578 (43.49)	253 (43.03)	325 (43.86)
**Remained in prediabetes**	233 (17.53)	120 (20.41)	113 (15.25)
**Progression to diabetes**	518 (38.98)	215 (36.56)	303 (40.89)
**Total**	1329	588	741

^a^Data are presented as n (%).

**Table 3** shows a univariable multinomial logistic regression analysis with unadjusted RRRs of variables used for the multivariable model.

**Table 3:** Prognostic factors associated with regression and progression in prediabetes over a 10-year follow‐up in a uni-variable analysis^a^.

	Regression to Normoglycemia	Progression to Diabetes
**Variables**		
**Age, years**	0.96 (0.95-0.97)	1 (0.99-1.01)
**Sex, female**	1.76 (1.60-1.94)	1.67 (1.51-1.84)
**BMI, Kg/m** ^2^	1.09 (1.06-1.12)	1.12 (1.10-1.15)
**SBP, mmHg**	1.007 (1.005-1.008)	1.006 (1.005-1.007)
**DBP, mmHg**	1.011 (1.009-1.013)	1.010 (1.008-1.012)
**Antihypertensive drugs**	1.57 (.88-2.80)	2.57 (1.51-4.38)
**Antihyperlipidemic drugs**	1.26 (.69-2.30)	1.89 (1.08-3.30)
**Familial history of T2DM**	2.58 (1.80-3.70)	3.29 (2.32-4.67)
**Waist circumference, cm**	1.009 (1.007-1.010)	1.008 (1.006-1.009)
**Glycemic status**		
**iIFG**	Reference	
**iIGT**	3.40 (2.65-4.56)	1.71 (1.26-2.31)
**Combined IFG/IGT**	1.10 (.77-1.59)	4.07 (3.03-5.46)
**TC, mmol/L**	1.17 (1.14-1.21)	1.16 (1.12-1.19)
**TG, mmol/L**	1.38 (1.29-1.48)	1.39 (1.30-1.49)
**HDL-C, mmol/L**	2.39 (2.07-2.76)	2.09 (1.80-2.42)
**Personal history of CVD**	2 (.96-4.12)	2.36 (1.16-4.78)
**Familial history of CVD**	2.64 (1.58-4.43)	2.39 (1.42-4.04)
**Smoking**		
**Nonsmoker**	Reference	
**Ex-smoker**	2.38 (1.50-3.76)	1.76 (1.09-2.86)
**Smoker**	2.57 (1.66-3.97)	1.85 (1.17- 2.94)
**Education**		
**<6 years**	Reference	
**6-12 years**	2.64 (2.11-3.30)	2.24 (1.78-2.82)
**≥12 years**	4.83 (3.11-7.50)	2.87 (1.80-4.57)

^a^Data are presented as RRR (95% CI). A total of 1329 participants remained in prediabetes as reference group. BMI, body mass index; CVD, cardiovascular disease; DBP, diastolic blood pressure; FPG, fasting plasma glucose; HDL-C, high-density lipoprotein cholesterol; iIFG, isolated impared fasting glucose; iIGT, isolated impared glucose tolerance; SBP, systolic blood pressure TC, total cholesterol; T2DM, type 2 diabetes; TG, triglyceride; RR, relative risk ratio.

We observed an age-related reversion to normoglycemia in the multivariable model. The regression probability decreases by 3% per year (RRR = 0.97; 95% CI, 0.95-0.99). Similarly, baseline combined IFG/IGT had a notable negative effect, and these participants had a 55% lower regression probability (RRR = 0.45; 95% CI, 0.29-0.70) compared with the iIFG participants. Women were 72% more likely to regress (RRR = 1.72; 95% CI, 1.18- 2.50). Higher education level (≥12 years) was positively associated with regression to normoglycemia (RRR = 2.10; 95% CI, 1.19-3.70) (**Table 4**).

**Table 4:** Prognostic factors associated with regression and progression in prediabetes over a 10-years follow‐up in a multi-variable analysis^a^.

	Regression to Normoglycemia	Progression to Diabetes
**Variables**		
**Age, years**	0.97 (0.95-0.99)	1 (.98-1.02)
**Sex, female**	0.72 (1.18- 2.50)	1.11 (.76-1.62)
**BMI, Kg/m** ^2^	0.99 (0.94-1.04)	1.10 (1.05-1.15)
**SBP, mmHg**	0.99 (.98-1.01)	1 (.99-1.01)
**DBP, mmHg**	0.99 (.97-1.01)	1.01 (0.99-1.03)
**Antihypertensive drugs**	0.95 (.49-1.82)	0.95 (0.52-1.76)
**Antihyperlipidemic drugs**	0.55 (0.28-1.08)	0.76 (0.40-1.42)
**Familial History of T2DM**	0.84 (0.55-1.29)	1.62 (1.07-2.45)
**Waist circumference, cm**	0.99 (0.98-1.01)	0.97 (0.96-0.99)
**Glycemic status**		
**iIFG**	Reference	
**iIGT**	1.43 (0.99-2.06)	1.08 (0.72-1.60)
**Combined IFG/IGT**	0.45 (0.29-0.70)	2.54 (1.71- 3.77)
**TC, mmol/L**	0.97 (0.81-1.15)	1.04 (0.87-1.24)
**TG, mmol/L**	0.98 (.84-1.14)	1.03 (0.89-1.20)
**HDL-C, mmol/L**	1.92 (.93-3.97)	.96 (0.45-2.04)
**Personal history of CVD**	1.31 (.60-2.88)	0.96 (0.44-2.08)
**Familial history of CVD**	1.02 (0.58-1.79)	.94 (0.53-1.66)
**Smoking**		
**Non-smoker**	Reference	
**Ex-smoker**	1.45 (0.84-2.50)	.94 (0.53-1.65)
**Smoker**	1.41 (0.83-2.38)	.96 (0.55-1.65)
**Education**		
**<6 years**	Reference	
**6-12 years**	1.19 (.81-1.76)	1.21 (.82-1.80)
**≥12 years**	2.10 (1.19-3.70)	1.72 (.95-3.09)

^a^Data are presented as RRR (95% CI). A total of 1329 participants remained in prediabetes as reference group. BMI, body mass index; CVD, cardiovascular disease; DBP, diastolic blood pressure; FPG, fasting plasma glucose; HDL-C, high-density lipoprotein cholesterol; iIFG, isolated impared fasting glucose; iIGT, isolated impared glucose tolerance; RRR, relative risk ratio; SBP, systolic blood pressure TC, total cholesterol; T2DM, type 2 diabetes; TG, triglyceride.

A higher BMI significantly increased the likelihood of developing diabetes from prediabetes. The progression probability increases by 10% for each BMI unit increase (RRR = 1.10; 95% CI, 1.05-1.15), whereas waist circumference had a negative effect on the progression to diabetes (0.97 [0.96-0.99]). The risk of progression was 62% higher for those with a positive family history of diabetes compared with those with a negative history (RRR = 1.62; 95% CI, 1.07-2.45). This predictor had a strong positive correlation with the development of diabetes, in contrast to regression to normal condition, which was inversely related to the combined IFG/IGT. Those with combined IFG/IGT were 2.5 times (95% CI, 1.71-3.77) more likely to develop diabetes than the participants with iIFG (**Table 4**).

As previously explained, due to the significant negative relationship observed between waist circumference and progression to diabetes, the researchers decided to investigate the correlation between waist circumference and BMI. They observed a high correlation, and as a result, sensitivity analysis was done. The relationship in the model containing BMI remained significant (RRR = 1.06; 95% CI, 1.02-1.10)(see **Supplementary Table 1**). Significance disappeared in the model when waist circumference was included (RRR = 1; 95% CI, 0.98-1.01) (see **Supplementary Table 2**). There was no change in the significance of other variables (see **Supplementary Tables 1, 2**).

## Discussion

The aim of the study was to simultaneously identify the determinants of regression to normoglycemia and progression to diabetes in individuals with prediabetes. In this population-based cohort study with a 10-year follow-up, we observed similar conversion rates of approximately 40% for progression and regression from prediabetes in participants, but with different predictors. A study on middle-aged participants with prediabetes showed that during a 10-year follow-up, the rates of regression to normoglycemia and progression to diabetes were about 23% and 30%, respectively (6). Another study among the middle-aged Swedish population with prediabetes reported a rate of regression of bout 36% during 8 to10 years (7). The KORA S4/F4 study of those aged 55 to 74 years in Germany found a reversion rate of 16.3% over 7 years of follow-up using an oral glucose tolerance test as the diagnostic criterion (8). The conversion rate varies based on the population characteristics, length of follow-up, and the definition used to define normoglycemia, prediabetes, and diabetes.

Our results showed that the factors that lead to regression from prediabetes to normoglycemia are not the same as factors that predict progression to diabetes. Age, sex, education level, and combined IFG/IGT predicted the regression. BMI, familial history of type 2 diabetes, and combined IFG/IGT are determinants of diabetes progression. The combined IFG/IGT were inversely associated with regression to normoglycemia and directly associated with the development of diabetes.

This analysis showed that younger age—independent of other factors—was related to a higher probability of regression to normoglycemia, which is in line with previous studies (1, 9). Aging is an inevitable risk factor for insulin resistance (10, 11). To reestablish the normal state, identification and intervention at younger ages may be considered. In this cohort, women had a higher probability of regression to normoglycemia. This finding may reflect a higher use of health care services and health awareness among women. A previous study (12) found an association between female sex and regression to normoglycemia, whereas other studies did not (13, 14). However, a study reported that women had a higher insulin secretion index (15). It is well documented that diabetes complications and burden vary between the sexes (16). However, there is not much proof of this problem in the stage of prediabetes. Previous studies have reported that women with prediabetes have a higher burden of cardiovascular disease as a complication of diabetes than their men counterparts (17). All of the aforementioned information suggests that among people with prediabetes, gender-related factors may need to be taken into account before diabetes actually develops. Age and sex are nonmodifiable factors that were associated with regression to normal glucose levels; however, they can be valuable for screening and intervention programs. This study found no correlation between conversions and blood pressure or dyslipidemia (low HDL-C and high cholesterol levels). Measures of lipid metabolism in relation to glycemic status have only been investigated in a small number of previous studies, and the results have been inconsistent (6, 18). In their study on 1610 participants with prediabetes, Ahmadi et al (19) showed that rising the trend of HDL-C was an independent risk factor for conversion to diabetes 9 years before the incidence of diabetes. In our study, although there were some associations between lipid measures and both regression and progression, this association disappeared after adjustment with other possible predictors. Based on these results, it appears that additional research may be required to examine how lipid components, which are frequently utilized in clinical laboratories as metabolites, contribute to the onset of diabetes. Lipid-lowering medication was associated with an increased risk of progression to diabetes in univariable analysis (RRR = 1.89; 95% CI, 1.08-3.30) but decreased the regression to diabetes by about 2-fold with a borderline significance (RRR = 0.55; 95% CI, 0.28-1.08). This outcome is expected given that statins make up the majority of antihyperlipidemic medications and that they raise the risk of dysglycemia. Our findings regarding BMI and waist circumference mostly agree with those of previous studies on the progression toward diabetes. In line with our findings, previous studies from India and South Africa did not detect any association between waist circumference and progression to diabetes (20, 21). However, weight gain, particularly visceral fat accumulation, could increase impaired insulin signaling, leading to insulin resistance and increasing the risk of progression from prediabetes to diabetes (22). In our study, this was shown with BMI.

In addition, our findings about the parental history of diabetes are consistent with findings from previous studies. Although the increased risk of progression to diabetes among those with a family history of diabetes shows some genetic effects, it may also indicate that individuals with a family history of diabetes are more likely to have their glucose level tested (23), and that a family history of diabetes probably affects an individual’s knowledge of having diabetes (24).

Diabetes risk is increased by both IFG and IGT, and it is increased by the two together more than by either one alone. This is consistent with the concept that any rise in glucose is not benign and reflects an endocrine pancreatic defect. The annual incidence of diabetes in people with IFG or IGT varied from 5% to 10%. Compared with normoglycemic people, the meta-analyzed relative risk and 95% CI for diabetes was 5.52 (3.13-7.91) in people with iIGT, 7.54 (4.63-10.45) in people with iIFG, and 12.13 (4.27-20) in people with both IFG and IGT (25). With the iIFG group chosen as the reference group in this study, we demonstrated that iIFG and iIGT had no differences in the progression to diabetes, but iIGT had a higher likelihood of regressing to normoglycemia with a borderline significance (*P* = .054). Insulin resistance in subjects with IFG is due to increased hepatic insulin resistance while in subjects with IGT it is related to the increased insulin resistance in skeletal muscles. However, there is a strong association between increased insulin resistance in liver and skeletal muscles. In both kinds of pre-diabetes, insulin resistance combined with β-cell dysfunction would be responsible for the increased risk of type 2 diabetes. ***(Abdul-Ghani MA, DeFronzo RA. Pathophysiology of prediabetes. Current diabetes reports. 2009 Jun;9(3):193-9.)***

Regression to normoglycemia was more likely to occur in participants with higher education levels. Education level is associated with income, occupation, and physical activity. Education also improves the willingness to seek health information and encourages healthy lifestyle behaviors. The inverse association between education level and diabetes and obesity has been supported by previous studies (26, 27).

In terms of possible clinical and public health significance of our findings, since identifying high-risk populations is considered a critical issue in diabetes prevention and intervention programs, pre-diabetes is an appropriate state in which high-risk individuals could be identified and followed for appropriate interventions. Therefore, identifying high-risk pre-diabetes people who progress to diabetes can help us carry out effective interventions to prevent diabetes, and even better control of these risk factors can increase the regression to normoglycemia.

### Strengths and limitations

The population-based longitudinal study design, multiple measurements from both clinical and paraclinical sources, including tests and questionnaires, repeated blood sampling, extensive follow-up, and use of an analysis that takes into account all outcomes simultaneously are the main strengths of our study. The loss of follow-up is a limitation in our study, as it is in any cohort study, which we tried to resolve *via* imputation. Another limitation is the definition of diabetes and normoglycemia, which was determined by a single blood glucose measurement; however, this is common in epidemiological studies. The diagnosis of diabetes in clinical practice is based on at least two measurements of hyperglycemia and using one measurement in epidemiological studies makes the results unreliable. In our study the fluctuation of glucose level could be on both sides, i.e toward diabetes or normoglycemia, so although it may decrease the reliability of the results, a measurement bias is not plausible. Furthermore, although there is a growing body of evidence describing how the types of prediabetes are physiologically different, because of the low sample size we could not separate the analysis based on the type of prediabetes. Nevertheless, the likelihood ratio test did not show a significant interaction between the type of prediabetes and different predictors.

## Conclusion

The magnitude of regression to normal glucose levels was the same as progression toward diabetes. We did not aim to investigate the reasons why people with prediabetes progressed to diabetes and regressed to normoglycemia, however, we did demonstrate that different factors can predict these related outcomes. Factors associated with regression to normal glucose levels were age, sex, and education level, and factors associated with progression to diabetes were BMI and familial history of type 2 diabetes. The combined IFG/IGT had a notable significant relationship with both, which indicates the major determinant role for prediabetes transitions.

In diabetes preventive and intervention programs, identifying high-risk people is thought to be a challenging task. It seems that prediabetes is a state in which high-risk populations should be identified, and essential interventions should be done. Identification of high-risk prediabetes individuals who go on to develop diabetes is crucial for effective diabetes prevention. Prediabetic individuals may progress to normoglycemia if these risk factors are better managed.

## Data availability statement

The raw data supporting the conclusions of this article will be made available by the authors, without undue reservation.

## Ethics statement

The studies involving human participants were reviewed and approved by the ethics committee of Shahid Beheshti University of Medical Sciences (Ethics approval reference number: IR.SBMU.ENDOCRINE.REC.1400.113). The patients/participants provided their written informed consent to participate in this study.

## Author contributions

The authors confirm contribution to the paper as follows: study conception and design: FH, FA, and DK. Data collection: FH, FA, and DK. Analysis and interpretation of results: ZA, KK, and DK. All authors contributed to the article and approved the submitted version.

## Funding

The main project has been funded by Shahid Beheshti University of Medical Sciences.

## Conflict of interest

The authors declare that the research was conducted in the absence of any commercial or financial relationships that could be construed as a potential conflict of interest.

## Publisher’s note

All claims expressed in this article are solely those of the authors and do not necessarily represent those of their affiliated organizations, or those of the publisher, the editors and the reviewers. Any product that may be evaluated in this article, or claim that may be made by its manufacturer, is not guaranteed or endorsed by the publisher.
