# Supplementary material for: Are the determinants of the progression to type 2 diabetes and regression to normoglycemia in the populations with pre-diabetes the same?
Source: Front Endocrinol (Lausanne). 2022 Oct 7;13:1041808. doi: 10.3389/fendo.2022.1041808 (PMC9585180; doi:10.3389/fendo.2022.1041808)
Supplement: Supplementary file 1 [file Table_1.docx]

Table 1 – Prognostic factors associated with regression and progression in prediabetes over 10 years of follow‐up.  Relative Risk Ratio and 95% confidence intervals (RRR; 95% CI) from multinomial logistic regression multivariable model with the 1,329 participants with stable remained in pre-diabetes as reference group (results of the sensitivity analysis including BMI).

|  | Regression to normoglycemia | Progression to diabetes |
| --- | --- | --- |
| Variables | **RRR (95%CI)** | **RRR (95%CI)** |
| Age (years) | .97 (.95-.99) | 1.00(.98-1.02) |
| Sex, Female | 1.73(1.19-2.51) | 1.10(.75-1.60) |
| BMI (Kg/m2) | .99 (.95-1.03) | 1.06(1.02-1.10) |
| SBP (mmHg) | .99(.98-1.00) | .99(.98-1.00) |
| DBP (mmHg) | .99(.97-1.01) | 1.00(.98-1.02) |
| Antihypertensive drugs | .93 (.49-1.78) | 1.06(.58-1.95) |
| Antihyperlipidemic drugs | .55 (.28-1.08) | .75(.40-1.40) |
| Familial History of T2DM | .84(.55-1.28) | 1.62(1.07-2.44) |
| Glycemic status |  |  |
| iIFG | Reference |  |
| iIGT | 1.42(.98-2.05) |  |
| Combined IFG/IGT | .44(.28-.69) | 2.41(1.63-3.56) |
| TC (mmol/L) | .96(.81-1.14) | .98(.83-1.17) |
| TG (mmol/L) | .98(.84-1.14) | 1.02(.88-1.18) |
| HDL-C (mmol/L) | 1.89(.93-3.84) | .811(.38-1.69) |
| Personal History of CVD | 1.32(.60-2.89) | .97(.45-2.09) |
| Familial History of CVD | 1.02(.58-1.79) | .93 (.53-1.65) |
| Smoking |  |  |
| Non-smoker | Reference |  |
| Ex-Smoker | 1.45(.85-2.49) | .83 (.48-1.45) |
| Smoker | 1.42(.85-2.38) | .84(.49-1.43) |
| Education |  |  |
| < 6 years | Reference |  |
| 6-12 years | 1.18(.80-1.75) | 1.14(.77-1.68) |
| ≥ 12 years | 2.08 (1.18-3.67) | 1.58(.88-2.82) |

Table 2 – Prognostic factors associated with regression and progression in prediabetes over 10 years of follow‐up.  Relative Risk Ratio and 95% confidence intervals (RRR; 95% CI) from multinomial logistic regression multivariable model with the 1,329 participants with stable remained in pre-diabetes as reference group (results of the sensitivity analysis including waist circumference).

|  | Regression to normoglycemia | Progression to diabetes |
| --- | --- | --- |
| Variables |  |  |
| Age (years) | .97(.95-.98) | 1.00(.98-1.01) |
| Sex, Female | 1.73(1.19- 2.51) | 1.27(.88-1.84) |
| SBP (mmHg) | .99(.98-1.01) | .99(.98-1.00) |
| DBP (mmHg) | .99(.97-1.01) | 1.00(.98-1.02) |
| Antihypertensive drugs | .92(.48-1.76) | 1.16(.63-2.10) |
| Antihyperlipidemic drugs | .55(.28-1.08) | .75 (.40-1.40) |
| Familial History of T2DM | .83(.55-1.27) | 1.65(1.09-2.48) |
| Waist circumference (cm) | .99(.98-1.01) | 1.00 (.98-1.01) |
| Glycemic status |  |  |
| iIFG | Reference |  |
| iIGT | 1.43(.99-2.07 | 1.02(.69-1.50) |
| Combined IFG/IGT | .44(.28-.69) | 2.36(1.60- 3.48) |
| TC (mmol/L) | .97(.82-1.15) | .97(.82-1.15) |
| TG (mmol/L) | .98(.84-1.14) | 1.02(.88-1.19) |
| HDL-C (mmol/L) | 1.95(.96-3.98) | .75(.36-1.58) |
| Personal History of CVD | 1.32(.60-2.89) | 1.01(.47-2.16 |
| Familial History of CVD | 1.01(.57-1.77) | .96(.54-1.70) |
| Smoking |  |  |
| Non-smoker | Reference |  |
| Ex-Smoker | 1.48(.86-2.53) | .80(.46-1.39) |
| Smoker | 1.43(.85-2.41) | .82(.48-1.40) |
| Education |  |  |
| < 6 years | Reference |  |
| 6-12 years | 1.20(.81-1.77) | 1.08(.74-1.59) |
| ≥ 12 years | 2.12(1.20-3.72) | 1.44(.81-2.56) |
